# The Operative Treatment of Appendicitis

**Published:** 1891-12

**Authors:** Thomas S. K. Morton


THIRD PAPER.

### THE OPERATIVE TREATMENT OF APPENDICITIS.

By THOMAS S. K. MORTON, M. D.

MR. PRESIDENT, LADIES AND GENTLEMEN: Since being requested by the directors a few days since to open the discussion of the Operative Treatment of Appendicitis, I have taken a glance through the literature of the subject, in order to offer, as it were, a consensus of opinion regarding the present status of the subject, as well as to draw conclusions from such personal experience as has fallen to my lot in this direction. Now I find myself embarrassed by the necessity of limiting my remarks to the few moments which are at my disposal, and to crowd into them even bare mention of the most salient facts. Hence, much must be entirely omitted and other points given scant attention.

The discussion being limited to operative treatment, pathology and diagnosis—perhaps the most interesting branches of the subject, even to surgeons—are not to be touched upon except incidentally. But I cannot refrain, in passing, from saying, that as the ratio of appendicular to cecal inflammatory affections is probably 100 to 1, hence that *differential* diagnosis in diseases of this region which is usually impossible prior to surgical interference, is neither necessary or important, as operative procedures up to the point of establishing diagnosis are identical for all affections of the cecal region. Again, I would condemn, without qualification, needle explorations as an aid to diagnosis. The procedure is inherently dangerous, and will furnish no indication that cannot otherwise be obtained.

The number of cases of appendicular disease discovered when we are upon the outlook for them, is astonishing. A large proportion of peritonitis cases in males, and especially in children, arise from this disorder; and in all cases presenting abdominal pain, whether acute, chronic, or recurring, no matter where referred, we should think of and examine for possible appendicitis. I have come to be very skeptical of such conditions as are described as abdominal "cramps," "colic," etc., particularly when of frequent recurrence. Curious as it may appear, yet it is a fact that the great majority of the profession are only now beginning to recognize cases of appendicitis and its consequences as such. Formerly the affection was almost universally diagnosticated as anything except itself. But just in proportion as the disease continues to be more certainly recognized, so surgeons are more early operating upon cases which demand interference, and, as a consequence, the mortality from the disease, as well as from the operation, is very rapidly on the decline.

Keen has said that "the first indication in appendicitis is to call a surgeon," that the physician, who almost invariably first sees the case, and the surgeon, may together watch the case, and if operation becomes necessary, interference may be prompt and well timed; while the surgeon will have the great advantage of being already familiar with the case, and not disposed to delay the operation that he may acquire such familiarity. Again, Mynter has well said that "we are utterly unable to judge correctly from symptoms alone of the extent and severity of appendix lesions, and for this reason alone abdominal section is and must be the safest method of treatment" in many cases.

*When shall we operate?* Judging from the cases that I have observed, and from the writings of others, I would formulate as a good working rule: To operate not later than the third day of disease, if the patient up to that time has failed to markedly improve under rest, restricted diet, purgation, and topical applications. Especially should this rule be adhered to in cases where we have failed to move the bowels—these are apt to be the fatal ones. Further than this, we should invariably operate as soon as the presence of pus is assured; when peritonitis is developing or spreading; when signs of sudden rupture of an abscess into the peritoneal cavity appear; and where septicemia from septic absorption is taking place. In children, operation must often be performed earlier than in adults, as with them the malady is more speedy in development, more fatal in tendency, and shows a greater proclivity to involve the general peritoneum.

But let me emphasize the point that *pain* is not a reliable symptom (especially when opiates have been administered) from which to judge as to whether the patient is better or worse; most weight should be given to the strength, temperature and condition of the bowels, stomach and general abdomen.

Mr. Treves urges that operation shall not be done until the fifth, sixth, or later day. But from my reading and experience I think this is too late. He argues thus, because few deaths occur before the fourth or sixth day. These cases, however, really begin to die the third, fourth, or fifth day, although death may not actually take place before the sixth or later day, when the possibility of benefit from operation has passed. If the case is progressing well and operation is being postponed, it should be watched and observed frequently and most carefully, for we cannot predict at what moment an appendix abscess may perforate into the peritoneum, or other dangerous complication arise, that will instantly demand operation.

If the case is operated upon early, the chances of recovery, as a rule, are exceedingly good. The mortality of appendicitis during the first forty-eight hours is almost *nil*, and the operative death-rate at that time is equally low. Later both rates increase, but the former much more rapidly than the latter. The patient, in this disease, is generally strong and well up to the moment of seizure, at which time the danger of operation *per se* is at the minimum. Such mortality as results in operations for appendicitis, has been mainly incident to undue delay. When physicians and surgeons generally have learned definitely to recognize such cases as are operative at a time before the vital forces have been too much sapped or dangerous complications have arisen, then will the mortality rate of both disease and operation remain steadily at a low figure.

Then, again, the local conditions, from an operative standpoint, are much less serious in the early stages. We have at first simply a swollen appendix, with infiltration, and, perhaps, a few adhesions. We then do not have to deal with fetid abscess, foul surroundings, and sloughing tissues, which may have given rise to intestinal gangrene and other complications, as well as to the impossibility of securing primary union of the wound. Hernia is more common as a sequel in cases where the operation is performed late, and where the surroundings are gangrenous, and we can only secure healing by secondary intent.

The cry of every writer is for earlier operations. I have found no surgeon who regrets having operated early, but almost all mourn cases that were operated upon too late. No case appears where a mistake in diagnosis has been made, despite the awful array of affections which has been drawn up as liable to render uncertain the recognition of appendicitis. On the other hand, very many cases opened with the expectation of finding other disorders have proved to be appendicitis.

*Who shall operate?* The operation for appendicitis may prove to be the most easy; but it is never trivial, often trying, and sometimes even baffling the skill of the very best abdominal surgeons. Hence, he who undertakes operation for the removal of the appendix for disease should be equal to dealing with any of the complications and emergencies of abdominal surgery. There is scarcely a complication which occurs in abdominal disease, that may not be met with in operations on the appendix. If a man knows only how to reach the appendix, it is not enough; he must be able to cope with any accident or emergency that may arise. Therefore, he must have had training in general abdominal surgery.

*How shall we operate?* There are two classes of cases to be dealt with. One, the acute, where there is perhaps abscess, perforation, or general peritonitis; and, second, those where operation is undertaken in the interval between acute attacks, as a prophylactic measure. The indications for the latter will be considered separately further on.

The preparations for the operation are usually of a hurried nature, on account of the active nature of the disease and the sudden determination that operation has become imperative. Previous purgation, if successful, will make the chances of recovery much more bright, no matter during what stage of the disease operation is performed. Cases where the bowels have been kept open from the outset of attack are always most favorable. Locally the abdomen should be cleansed as for any other operation.

All writers now agree that the incision should be lateral. Median incision is only permissible when diagnosis from other abdominal disease is not clearly made out, as where we have had suddenly developed, violent peritonitis arise without obvious cause. Even should the median incision have been made, and the affection prove to be appendicitis, especially if septic, a lateral incision should still be resorted to, for it is exceedingly difficult and dangerous to drain septic appendicitis cases through a median incision, and often it is impossible to deal with complications, or with the appendix itself, except by the more direct route. I am of the opinion that almost any complication arising from appendix or cecal disease, can best be dealt with through the lateral incision. No writer has regretted making the lateral incision, although many have regretted entering through the linea alba.

This incision should be about three or four inches in length, and terminate one inch and a half above Poupart's ligament. It should be carried down to its full extent through the right linea semilunaris until the peritoneum is reached, avoiding, if possible, the epigastric artery, which normally would be situated to the inner side of the lower extremity of the wound. I have seen serious secondary hemorrhage from division of this artery. Having reached the peritoneum, if one does not at once get into the abscess cavity, we must exercise great caution not to open the gut by mistake. Sometimes adhesions will be found binding intestine to the peritoneum in the line of incision, and in these cases it is well to go at once to the lower or upper extremity of the wound, get into the general peritoneal cavity, and work upward or downward, as the case may be, to the cecum, when all adhesions can be separated by the finger or knife, and the peritoneum opened to the full extent of the external incision. Of course, the incision should be increased in size if there is any difficulty in getting into the peritoneal cavity, or subsequently if difficulty arises in any manipulation from lack of working room. But, as a rule, the smaller the incision the better, because of the less risk of subsequent hernia. The head of the colon is then sought out. If now it is found difficult to determine the site of the appendix, the longitudinal muscular bands of the colon may readily be followed down to their termination in the root of the appendix. Then by careful manipulation one can usually trace the appendix, even through a mass of dense adhesions, and dissect it out. As a rule, in acute cases the organ will be found more or less free in the cavity of an abscess, with its tip, perhaps, adherent to omentum or bowel. The appendix is to be dissected out with the finger, and often we do not see it until it is brought out of the wound ready to be ligated off. This manipulation closely corresponds to the modern one of removing the uterine appendages.

Now, what shall be done if the appendix is found to be bound down by a dense mass of adhesions, and if it would take a long dissection and endanger life from the time required to complete the operation? Under these circumstances I would advise that the appendix be left alone rather than run any great risk of the patient's life to complete an ideal operation. We are often compelled to operate to save life, and that alone, even if we do run the risk (as of leaving the appendix) of recurrence. I do not regard the operation as complete in any case unless the appendix is removed, and we should never hesitate to dissect out or remove the organ simply for fear of opening up the general peritoneal cavity.

Cases of recurrence, with great violence of symptoms, are upon record, where operation had been performed and the appendix not removed. Here, again, we have a parallel with the removal of the uterine appendages. Who considers that he has done a complete operation when he simply drains a pyosalpinx? Yet there is a small (but constantly decreasing) proportion of these cases that must be so treated rather than endanger life by prolonging operation, shock, and anesthesia.

If the appendix can be excised, the question arises as to how we shall deal with it after separating all adhesions. In septic cases it will be found usually impossible to invaginate the stump, after cutting away the appendix, into the cavity of the cecum and approximate peritoneum to the remaining opening. Where we operate between attacks, the appendix, as a rule, can be dealt with in this manner and the invaginated stump retained by a few Lembert sutures, approximating the surfaces of the cecum over the aperture. When, however, the organ and its surroundings are swollen and gangrenous, the conditions are such that it is generally impossible to invaginate the stump. It has seemed quite sufficient in these septic cases to ligate the appendix a quarter of an inch from its root with strong silk, and then cut off both the appendix and the ligature ends. But ligatures will neither become absorbed or encapsuled where septic conditions are present, and I have seen the threads coming out of the wound months afterward from persistent sinus or by ulceration. So it occurred to me that we might resort to the old surgical procedure of leaving one end of the ligature hanging out of the wound. That experiment I am now trying in a recent case. Chronic ligature sinuses assist in the production of hernia by interfering with solid union.

Frequently the appendix will be found with a mes-appendix. This should be ligated *en masse* or in sections, and cut away from the appendix. Then the appendix is ligated at its base and removed. Removal of the appendix is almost universally recommended, but Mr. Treves has simply straightened an appendix which he found angulated by adhesions and left it in the wound. Mr. Tait has practised in more than one case splitting open the appendix and inserting a fine drain-tube into it. From these instances it will be seen that there exists in some minds an almost superstitious fear of removing the appendix. Certainly no sentiment can exist concerning the ablation of the appendix such as there is in regard to the ovaries and Fallopian tubes. Having the appendix once in hand, it does not add to the dangers of the operation in the least degree to remove it, while recurrence of the disease is thereby rendered impossible.

Occasionally the appendix is found to have sloughed off at its root, leaving a ragged opening into the cecum. In one or two cases the edges of the opening thus left have been inverted and closed successfully by Lembert sutures. In others the wound was left entirely open and packed with gauze; an intestinal fistula or artificial anus formed, but in time closed spontaneously. Yet another required a subsequent operation and Lembert sutures before it was cured.

Some surgeons recommend that in septic cases a little flap of peritoneum be sewed across the stump, or that it be tucked under a bit of omentum. I can see no advantage in this. It prolongs the operation and does no good, while by so doing we risk the formation of a secondary abscess pocket. Very many appendix stumps have been simply dropped into the wound again after ligation; fecal fistula did not form, and the wound closed satisfactorily.

Any portions of gangrenous omentum presenting in the wound should also be ligated beyond the junction with healthy tissues and cut off. Any small openings into the peritoneal cavity may next be sewed up carefully if the general peritoneum does not require drainage.

Then in regard to irrigation: if the general peritoneal cavity has been opened extensively, or if it is septic, it should be thoroughly washed out through the lateral incision. If it has not been involved, the abscess cavity and wound alone should be irrigated. Under the latter circumstance we may employ a strong bichloride solution, but if the peritoneum is to be flushed, nothing but water should be used.

If the general peritoneum has been septic or extensively opened or manipulated, it is essential to use drain-tubes to the base of the pelvis. The ordinary straight glass tubes do not answer well, and rubber is not satisfactory. Here I have a collection of angulated and curved glass tubes, most of which have been used with great satisfaction in appendix cases. The angle makes it possible to get the tube to fit well over the brim of the pelvis, yet not to project awkwardly from the lateral wound. By attaching a few inches of rubber tubing to the end of the ordinary cleansing syringe, the bent tube can readily be cleaned.

The suturing of the wound is especially important if the case is *not* a septic one. Then the tissues should be sutured layer by layer; this gives the best assurance of firm primary union and the avoidance of hernia. If, however, the wound is septic, and drainage or packing is employed, secondary union is inevitable. But I would still urge that the wound be as carefully sutured as possible in all cases, leaving ample room for exit of the drain-tube or packing. And I might say, in passing, that simple packing with strips of double cyanide or iodoform gauze will be found to answer all purposes of drainage in cases where the general peritoneum does not also require drainage.

Some surgeons advise using no stitching in septic cases, but simply packing of the entire wound with gauze. But by suturing we can usually secure primary union in a portion of even a foul wound, and temporary stitching has appeared to give a certain anchorage and support to the subjacent intestines, which, when the sutures are removed, is more or less retained. The stitches, of course, are to be removed, one or more at a time, when swelling, infiltration, tension, or deficient drainage, becomes apparent. Strips of adhesive plaster should be employed to give the wound support and approximation during granulation.

Complications such as gangrene of intestine or mesentery, must be dealt with upon general principles of abdominal surgery. If intestinal obstruction complicates the case, the site of obstruction should be ascertained, and the condition relieved, if possible, before closing the wound. Cases in which obstinate constipation has existed up to the time of operation, should be examined during its performance for possible obstruction.

Should peritonitis develop subsequent to operation, and not speedily yield to active purgation, the wound must be reopened, and the abdominal cavity irrigated thoroughly and drained. Continued obstruction could probably be best dealt with through a new median incision rather than through the original wound.

As soon as the patient comes out of ether, if the bowels have not been well emptied before operation, it is my custom to at once begin the administration of one-eighth grain doses each of calomel and podophyllin, at twenty-minute intervals, until purgation is accomplished. This usually takes but very few hours. Later, salines may be employed if required.

Full strength peroxide of hydrogen solution has given me great satisfaction for cleansing and washing the wound cavity when suppuration commences and sloughs are forming—it greatly facilitates the separation of the latter.

Persisting fecal fistula usually close spontaneously in time. Should they not, then reopening of the parts several months later, and suturing of the cecal or other opening with Lembert's sutures, is indicated, and has proved successful in several instances.

In conclusion, let me say a word in regard to operations undertaken in the interval between acute attacks, or what may be termed *prophylactic operative treatment.*

The indications for this measure are: Constantly recurring attacks (usually indicative of the presence of a foreign body in the appendix), which interfere with the individual gaining a livelihood, or render his life a constant burden, worry, and expense to him; also, where recurrent attacks have taken place in those, as seamen, hunters, explorers, etc., who are liable to be again attacked when they may be out of reach of adequate surgical aid. In this class of patients, operation during quiescence of the disease should be considered, and, perhaps, urged by the medical attendant. In most other cases, I do not think excision of the appendix should be often attempted in the quiescent period. We should rather counsel delay until the onset of the next acute seizure, when we can conscientiously urge the removal of the offending organ at once—that is, on the first or second day. This advice is given principally because of the great difficulties and dangers frequently encountered in operating during the intervals of attack when the adhesions are extremely dense. In fact, patients have died as a result of the long time required to complete the operation, because of the elaborate dissection required to free the appendix from its matrix of densely organized adhesions. In several instances the very best operators have been compelled to abandon these operations in the intervals of attacks, not only without having been able to remove the appendix, but also without having been able to discover the organ in its bed of adhesions.

### DISCUSSION.

Dr. WILLIAM PEPPER: I scarcely think that I need say much, for the subject as presented is so largely one of operative technique, that the views of a purely medical clinician possibly are scarcely appropriate. Assuming that the subject under discussion includes all the acute inflammatory affections of the appendix, cecum, and pericecal tissues, much has been said to which I should take strong exception from the standpoint of a pure medical practitioner. I believe that if every case of appendicitis were operated on, the mortality would be ten-fold what it now is. For more than a quarter of a century I have been in the habit of seeing a great many cases of appendicitis every year. I base this statement partly upon the classical researches of Dr. Fitz, who has demonstrated more clearly than any other, that in a large proportion of cases of right iliac trouble the appendix shares in the disease, if, indeed, it is not the starting-point of the malady. Now, as a general rule, these cases recover under medical treatment, and remain permanently well afterward, no surgeon being associated in the treatment of the case. In no year during the past two decades have I failed to see a considerable number of cases of this kind, and the cases that have demanded operation, as contrasted with those which get perfectly well without operation, is probably at least as one to a score. I think the assertion that as soon as appendicitis is suspected the surgeon should be called in, is quite out of accord with the experience of physicians the world over. As I have said,

I think that the vast majority of cases, in first attacks at least, undergo resolution and terminate with some more or less permanent injury to the appendix, but without going on to the production of abscess, provided the treatment be instituted early and be kept up faithfully. In many of these cases there is early development of induration and fulness in the right iliac fossa, and in proportion as this appears early, is it likely that the case will run a favorable course, or, if later it develops signs of suppuration, it will admit of treatment by the simple Willard Parker extra-peritoneal incision. In proportion as the symptoms are violent, without localizing phenomena in the right iliac fossa ; is there danger that rupture of an abscess has occurred, to be followed by the development of general peritonitis. I am entirely at one with the speakers who insist on early operation where this latter condition exists. I have had the operation performed as early as thirty-six hours from the initial symptom, and have found suppurative peritonitis already present sorry to say that in this case there was a fatal result, as will sometimes happen in the hands of the most skilful operator. I think that the experience of all will confirm the statement that the operation is a grave one. The operation of laparatomy for disease of the appendix, whether it is exploratory or radicle, is not a trifling operation, and I have rather extensive records to show that it is an operation attended with a great deal of danger, even in the hands of the most brilliant operator. I should protest against the view that, as soon as the diganosis of appendicitis is made, an operation should be encouraged.

I believe that it is possible to note the time in a certain large proportion of such cases, when the symptoms indicate the spread of inflammation, and then I think that the operation cannot be too promptly performed.

The question of diagnosis remains, in spite of all the good work that has been done, a most difficult question. The McBurney point I believe to be largely without value, uncertain in its location on account of the very varying relations of the appendix, apt to be mistaken for points of tenderness due to wholly different causes, and apt possibly to be mistaken for sympathetic tenderness of nerve points in the abdominal wall. I, therefore, believe that this sign, from which much was hoped, will prove to have very little positive diagnostic value.

The rectal examination has seemed to me to be of very material value ; it is true not so early as we could wish, but in many operative cases I have found the roof of the pelvis altered as determined by a careful rectal exploration. I feel that I am wholly incapable of putting in words, nor do I know that this has been done, the exact differential diagnosis of the cases which demand early operation. While this is true, I would still urge the view that this does not justify the subjection of every patient with appendicitis to laparatomy. I trust that we shall learn to arrive at a more exact differential diagnosis. There is a combination of a certain history of the development of the case, which, taken in connection with the facies, the general symptoms, and the abdominal condition, as determined by external and by rectal examination, will, in the hands of an experienced clinician, serve in the great majority of cases as a basis for this diagnosis. It is difficult to state this in terms as precise as we should state the terms of a diagnosis of encysted pleurisy, but I think that those who have studied these cases will recognize a *tout ensemble* which admits of a diagnosis of those cases which should be subjected to early operation. I believe, on the other hand, that in the great majority of cases we are justified either by the mildness of the symptoms or the localizing tendency in the right iliac fossa in urging medical treatment, and this is further justified by the very frequent recovery of these cases.

Lastly, I shall say a word as to my entire opposition to operation in the majority of cases in the interval between recurring attacks. I think that medical records will show too many cases where thorough treatment, hygienic, dietetic, and medical, has been followed by complete cure. I have had so many such cases in which cure has occurred after a number of recurrent attacks, that the adoption of a general rule that where a patient has had two, three, or more attacks, he should be subjected to a grave operation like laparatomy, seems to be a dangerous postulate. I think it better to secure the consent of the patient to the performance of the operation, should alarming symptoms make their appearance in any attack, and then to persevere with carefully regulated medical treatment. There are cases, unquestionably, where the conditions of the patient, the fact that he may be attacked when out of reach of skilful surgical aid, make it necessary for the patient to decide between a change in his habits of life and an operation. These are exceptions, and it does not follow that a general rule that laparatomy should be performed in the interval between recurrent attacks of appendicitis, should be laid down.

Dr. Keen: I wish to take exception to what Dr. Pepper has said in reference to not calling in a surgeon in a case of appendicitis until operation is needed. I think that it is of the most urgent importance that the surgeon be called in not to do an operation, but for consultation, for his judgment rather than his knife — not necessarily to do a, laparatomy immediately — but for the purpose of being ready to deal intelligently and promptly with the conditions when the time for operation arrives. He should not be called in then, new to the case and unfamiliar with its features, and desiring, therefore, time to become familiar with it, unless the case is so serious that operation is evidently and instantly required. The surgeon should be with the physician the moment the diagnosis is made, not to do the operation then, but to be ready to do it the moment that it becomes necessary. I have seen cases lost, and have lost some myself, I am sure, from delay, from the natural unwillingness to plunge right in and do a laparatomy the moment we are called to see a case that really needs it, and yet from unfamiliarity is regarded as a doubtful case. We should have every point at our fingers' end and be familiar with the fluctuations of the symptoms. Then our aid will be much more valuable than if we are called in only when the emergency for operation has arisen. A plain case every one can read and decide quickly. It is the doubtful cases that need carefully weighed decision — a snap judgment on a sudden call is more apt to be wrong than right.

Mr. Thomas Bryant, of London (by invitation): I assume that the term appendicitis as here used includes all those cases which have been spoken of as typhlitis, pertyphlitis, and by other names, all of which have probably more or less connection with the appendix itself. Starting with that assumption, I at once proceed to the treatment of appendicitis. Here at the beginning, although a surgeon, I agree very strongly with the observations of Dr. Pepper. I am convinced that operative treatment is most valuable in appendicitis. I am equally convinced that delay in operating is the wisest course in the majority of cases. I should like to say in this place it seems to me that the authors are a little mixed in regard to the classification of these cases. They have included cases that are acute from the beginning, with cases that are not acute, that have a slow and steady course. The cases that have a slow and steady progress, that begin with localized pain is the right iliac fossa, accompanied with tenderness and soreness, less swelling without any very acute symptoms, are cases which you must feel can be dealt with satisfactorily without the surgeon's knife; I do not say without the surgeon's aid, but without the surgeon's knife.

Dr. Morton spoke strongly of the use in these cases of calomel and podophylin. Such statements rather startled me and I should have been glad to have had some evidence of its value given. I should prefer to follow the line of treatment suggested by Dr. Pepper and not give calomel and podophylin in frequently repeated doses. I would rely more upon rest, belladonna externally and opium internally, and diet, believing that by such means the bulk of the cases are permanently cured. In exceptional cases where these good results do not occur and graver symptoms appear, the swelling increases and symptoms of peritonitis develop, the surgeon's aid becomes of immense value, and certainly where these symptoms do appear and there is a steady progression toward the bad, it is, unquestionably, time for the surgeon to take a hand. In all acute cases I have no doubt as to the right of the surgeon to interfere. I have seen cases where within thirty-six hours after such acute symptoms it was necessary for the surgeon to expose the part and let out the inflammatory fluids, if not remove the appendix itself. To my mind these two classes of cases, which I have briefly described, fairly indicate the line that the surgeon should take: trusting very much to expectant treatment in the least acute cases, and surgically interfering early in the acute.

In reply to the question in regard to the propriety of operating, whether or not the surgeon is justified in operating between the attacks, my judgment would decide in the negative. In the majority of cases there is no second attack. If there is a second attack, it can be treated on the same lines as the first, only there is a tendency toward interference if the symptoms do not settle down rather rapidly. I say this because I am sure that I have seen many instances where things have settled down after a second attack without any further trouble. Because we have met with cases that after the second, third, fourth, or it may be the eighteenth attack, have at last come to the surgeon's knife, I think that we should not accept that as a decided evidence in favor of surgical interference. In fact, we must be governed by each case itself, and we should surgically interfere only when we find small chances of Nature terminating the case guided by medical skill.

Now we come to the operation. I am not sure that I am quite in accord with the authors of the papers. It is quite true that in doubtful cases of appendicitis — that is, cases in which you do not expect to find a great deal of pus or inflammatory fluid, the incision in the right semilunar line will probably be the best. In this way you come down readily on the cecum, and you are more apt to find the appendix. The majority of cases with which the surgeon has to deal are not quite in the stage to which I have referred. There is generally much more diffused swelling about the cecum, and that swelling gravitates backward and upward, sometimes toward the loin. I can recall a good many cases that I have opened where I was certain the swelling was about the cecum, but it was found backward toward the lumbar region. I can recall several instances in which my attention was drawn more to the lumbar region than to any other part, and it was only by going into the history that I concluded that the trouble was located in the cecum. The lateral incision is a good one in these cases, but it must be more lateral than the semilunar line. I have made my incision well back, corresponding to the line of the anterior superior spinous process and tending backward toward the loin. In this way you get well at the cecum and your finger can be readily passed into the iliac fossa. You can examine the part, you can drain the part well, and generally by the open treatment, not being too careful to stitch the wound, a good result takes place. I would say that in a large number of cases—my friends may say neglected cases—that an incision more posterior than the semilunar line would be the better one. The incision in the semilunar line should be reserved for cases that have not advanced to such an extent as I have just indicated. If there were time, I could give the Society many cases as illustrations of the truth of what I have said.

Another point to which I should like to allude, is the question whether or not these are all really cases of appendicitis. In at least three instances of cases which had presented a history of a cecal trouble, but in which death had resulted from some other cause, I have found cicatrices in the posterior part of the cecum, some distance from the appendix. In two cases that I have treated, the evidence pointed to the cecum as the seat of trouble. In one, a boy aged twelve years, I incised an abscess, and eventually a large orange seed escaped. I have no reason to believe that that could have come from the appendix. In the second case, a piece of bone, that had been swallowed, had evidently passed through the wall of the cecum and caused suppuration. These two cases presented all the features of typical appendicitis. They were dealt with in the way that I have stated, and both recovered. We must, I think, bear in mind that these cases are not all due to disease of the appendix, and that many of these may have no connection with it.

This brings me to another point, and that is, whether or not, under all circumstances, it is expedient to search very carefully for the appendix.

In these severe cases should we disturb the parts so much as is often absolutely necessary? We have had, tonight, good evidence of the difficulty of finding the appendix in some cases. I have always felt that in these cases we should do more harm than good if we searched too far for the appendix. I am satisfied with well irrigating the part and treating it by the open method.

Dr. Morton has mentioned hernia as following the operation. I have never seen this. That may be because the bulk of my incisions have been made posteriorly. I have done many of these operations, and have seen many others done by my friends, but I have never seen hernia as a result.

Dr. J. M. Baldy: It has always seemed to me that it was not so much a question of the diagnosis of appendicitis, as the differentiation between the operative and the non-operative cases. The diagnosis of appendicitis *per se* is extremely easy; at least, so I have found it. As far as symptoms are concerned, I know of only one that is of fixed value, and that is, constant, deep-seated pain in the right iliac fossa, with induration. I think in such a case there is little question but that there is inflammation in or about the head of the cecum, and, presumably in the majority of cases, in the appendix.

Mr. Bryant has spoken of cases where large foreign bodies have been discharged through an abscess, and claims they have come from the cecum. He offers no evidence of this, except the size of the body. I have seen the appendix sloughed off, leaving a sufficient opening in the cecum to admit the index finger, so that I cannot see that the size of the body indicates in any way that it came from the cecum, and not from the appendix.

I have been glad, and, at the same time, rather surprised to hear the McBurney point condemned. I believe that it is utterly worthless as a reliable point in the diagnosis. It is one of those attempts at refinement in diagnosis which are apt to lead only too many astray. I have tried to apply McBurney's point, but have failed in every case.

The rectal examination may be of value in many cases, but we have all seen cases— and Dr. Keen's is one in point—in which there is a small abscess high up, which could by no possibility be recognized by rectal examination. If the abscess contains many ounces of pus, it will generally extend downward to the pelvis, and may be felt through the rectum. There are, however, so many cases in which this cannot be done, that we can place no definite value on this method except in a limited number of cases.

I cannot help thinking that purgation is of distinct value when I see the great relief afforded to a man groaning with the most intense pain, simply from having a movement of his bowels. It may not be curative, but in every case, whether abscess is present or not, it gives great relief. I believe that purgation should always be used. At the same time, if the patient was suffering, I should not hesitate to use opium until the purgation had acted, or after it had acted in case of necessity. The amount required is not great, and will not interfere with the purgation. Those cases in which it is difficult or impossible to induce purgation, are going to do badly.

I know of no other intra-abdominal disease in which it requires more skill and practical experience to differentiate between those cases that should be let alone surgically, and those which should be operated on. I grant that the majority of cases of appendicitis get well without any operation. Again, there are certain cases that do badly from the beginning, and in which operation is clearly indicated. But, with certainty, there remains that large class of border-line cases, in which it is next to impossible to say whether pus is present or not. If there *is* pus, no one should hesitate. The operation for abscess is simple and easy. The abscess once opened, I do not think that in these acute cases any time should be lost in searching for the appendix. In trying to find the appendix, and even when found, in trying to remove it, the general peritoneal cavity will often be opened, and life will be lost where otherwise it would have been saved. Only one case, as far as I am aware, has been reported in which the abscess has been opened, and the appendix left, where a second operation was required for a severe recurrent attack.

I believe with Dr. Keen that the surgeon should be associated with the case from the beginning, although not necessarily to operate. When a physician is called in to operate, the tendency, if there is doubt—and doubt only too often exists—is to postpone the operation. If the surgeon has seen the case from the beginning, and studied the symptoms and knows the details, when the time comes he will have made up his mind whether or not to operate. If, however, the surgeon loses another twelve or twenty-four hours in hesitation, in addition to what the physician has already wasted, the patient may be irretrievably lost. The deaths after operation are due not so much to operation as to delay.

It is impossible to lay down any rule as to the time at which operation should be performed in any case, or in any class of cases. In some, the onset is so sudden and violent that it is impossible to come to any decision as to the seat of disease. This was the case in the patient reported by Dr. Keen, and in another instance I know of in New Jersey. In the latter case, it was not until a few hours before death that the symptoms were sufficiently marked to cause any alarm. This is often the history of cases in which the operation is postponed. The patient will be doing well until within a few hours of death, when the end comes suddenly, and the patient sinks rapidly.

Dr. Frank Woodbury: Although as a physician I look at this subject from the medical standpoint, I am in favor of operating. To save time, I may say that I heartily coincide in the statements just made by Dr. Pepper. I also endorse the remarks of Mr. Bryant, in which he anticipated what I had intended to say —that is, that each case must be studied by itself. I think that the surgeon and physician look at these cases a little differently. The surgeon is looking for general rules to govern him in the treatment of cases, while the medical man is more in the habit of individualizing his patients. Concerning the propriety of operation and the results to be anticipated therefrom, we all acknowledge that there is in some individuals a tolerance to operative interference and to suppuration that does not exist in others. Some will survive dangerous gunshot wounds; others will perish from a slight injury. There are probably points that will enable us to determine this difference in tolerance of different individuals, and, were they in our possession, should undoubtedly enter into the question of operation, but at the present time, unfortunately, we are not prepared to formulate these points in any given case of appendicitis which comes before us. Physicians who are accustomed to go into the history of the patient and to investigate the antecedents of the case, know that individuals who belong to families with a high physical standard, and who lead regular lives, are able to stand severe illnesses and operations, coming through them speedily and well, while others; having poor family history, bear very little surgical interference, and easily succumb to disease.

The question of the time of operation and propriety of operation really resolves itself into the query, "At what time does a case of appendicitis become a surgical case?" I would here raise my voice against the physician yielding to the temptation to put a hypodermic needle into an iliac abscess or swelling in order to make the diagnosis. As soon as he does that, he takes surgical responsibilities on his shoulders, and in no case are they likely to be more serious than in appendicitis. The physician should have sufficient surgical knowledge to determine when the time for operation has arrived, or, acknowledging his inability to decide this, he should secure the best obtainable surgical advice, not necessarily to operate, however, but to determine the propriety and proper time for operation, if found necessary.

Among the cases that come to my mind, three stand forth prominently. One was the first case which I saw, some eighteen years ago. A man in the lower walks of life continued at his work as a machinist, making no complaint, until one day he fell on the floor of the shop in a collapse, and was brought to the Pennsylvania Hospital. He had a feeble pulse, and a hippocratic face; the surface was cold, and, as he was dying, no attempt was made at diagnosis. He died in a few hours, and a post-mortem showed it to be a case of perforative appendicitis, with perforation and the usual foreign body. Here there was no question of operation. The patient did not seek medical advice, and there was no time for operation.

The last case I saw in my own practice occurred last Spring, in a patient whom I had attended at intervals for a number of years. I had attended him a year before with a light attack of appendicitis, and warned him that if he had a subsequent attack he should consider the question of operation. He did have a subsequent attack while away from the city, and on his return was attended by another physician for three months. The man improved and was about, but always felt a weight and pain in the right iliac fossa. He was then taken with acute obstruction of the bowels with intense pain, and finally I was sent for. In this case nothing that was given him produced a movement of the bowels. I may say that in this case Dr. Thomas G. Morton operated on the second day after I saw him, but the patient died five days later without a movement of the bowels. The bowels were probably matted together and gangrenous.

The third case that occurred to me is one operated on also by

Dr. Thomas G. Morton five years ago, which I have reported to the College of Physicians[1] and to this Society. I believe that Dr. Morton claims that this was the first case in this country where the correct diagnosis was made prior to operation of amputation of the appendix, and where the patient recovered. The patient has been well since the operation, although prior to it he had had a number of attacks. The patient is present tonight, and I should be pleased to show him to the Society. He is still wearing a light truss to protect a weak place in the abdominal wall at the lower portion of the incision. [The patient was exhibited.]

Dr. H. A. Hare: I rise for information rather than to discuss the surgical aspect of the papers which we have heard. Like Mr. Bryant, I am at a loss to know why calomel and podophyllin, the latter in such large amounts as one-eighth of a grain every twenty minutes, should be given after an operation for appendicitis. Podophyllin is the slowest acting purge in the Pharmacopeia, taking eight or twelve hours to produce an effect, as a rule; not only this, but these drugs act on the small bowel, high up, while the appendix is in the large bowel, low down. If saline purgatives were ordered, it seems to be a better treatment, for we have evidence of their great value. Even these are not without danger. I do not believe that a man can take one-eighth grain of resin of podophyllin every twenty minutes until he is purged without producing much intestinal griping and pain. Anstie pointed out the fact that podophyllin was a distinct irritant, particularly to the small intestine.

Dr. De Forest Willard: Mr. Bryant has said that he has not seen hernia follow this operation. A boy came into my office today on whom I operated two years ago. He did perfectly well for a year, when, on attempting to lift a heavy body, the bowel protruded through the center of the cicatrix. He had worn a bandage and a truss, but I put on a heavier truss, which relieved him for a time. He returned in four months; the pressure of the truss had produced a large slough, and he came near having a perforation of the bowel. The ulcer finally healed. He now has at the outer angle of the wound a second small hernia, and at the inner angle there is a slight tendency to protrusion. The wound was a large one. The boy was in extremis at the time of operation, and there was an enormous accumulation of pus.

In regard to deep-seated pain and induration in the iliac fossa

1. Case reported in Proceedings of the College of Physicians of Philadelphia, vol. vii.

as a diagnostic sign, I have seen cases in whom there was not a particle of local pain or of induration.

The boy already mentioned had no such symptoms. He had been kicked at the umbilicus, and the pain was chiefly at that point, yet the abscess was in the iliac region. The appendix was open, and a small mass of feces had escaped. There was an enormous accumulation of pus extending down into the pelvis on one side. The appendix was removed, and the opening stitched.

Dr. M. F. Kirkbride: In the past year and a half I have had four cases, but shall speak only of one. I shall first refer to the history of the case as given in a letter to the previous attendant. The physician was called on Thursday, April 3d. The patient had been constipated for one day. It was at first thought that the case was one of typhoid fever, as the father had recently recovered from this disease. Calomel was given, but no action secured. Citrate of magnesia was given with the same result. The pain and tenderness in the right iliac region increased. There were some tympanites. Injections of soap and water with a few drops of turpentine were practised with no result. Salts in one-drachm doses were then given without effect. Vomiting began. It became apparent that it was a case of appendicitis with obstruction of the bowels. Injections given on Saturday morning were not retained. On Saturday a surgeon was called in consultation. Operation was decided on, but as the surroundings were not suitable the family was advised to have the patient admitted to a hospital. This they agreed to do, but at 3 P. M. decided not to do so. The physician in charge then declined to have anything more to do with the case.

I was called to see the patient on Sunday evening, at ten o'clock. The temperature was 99.5°, the respiration, 36. No pulse at the wrist. The heart-beats, 130. He vomited everything, and for several days had had stercoraceous vomiting. I gave hypodermics of morphine and atropine, and afterward hypodermics of strychnine. After he had re-acted somewhat, I put him in the knee-chest posture, and gave an enema of sulphate of magnesia, turpentine, glycerine, and warm water, and gave whiskey and turpentine by the mouth. I also gave for several hours sulphate of magnesia in one-drachm doses. The first two doses were rejected, but afterward there was no vomiting whatever. In three hours I had the tumor removed and the boy sleeping comfortably, and after that he got along nicely. On the sixth day after I was called, a slough passed from the bowel. This was three inches in diameter. The case then went through that of a regular case of typhoid fever, as far as the temperature was concerned, and even showed the eruption. The diagnosis of appendicitis was made by two physicians and an eminent young surgeon.

Dr. Joseph Hoffman: McBurney's point has been condemned, but the reasons have not been given. The position of the appendix varies. You cannot lay your finger on any special point and say that there the appendix should be found. We must remember that the appendix revolves in three planes, and that, therefore, it may have three systems of revolution. We cannot expect to find the appendix always in the same position. This anatomical fact forever blots out McBurney's point.

In reference to purgation, I had a case, of which I shall not recite the points, in which the use of calomel and opium turned out beautifully, so far as the apparent curative effects are concerned. Dr. Wheeler was treating a case of appendicitis with opium for some days without benefit. He then called me in and I brought Dr. Price. He was then purged with calomel after the opium treatment, and the pain entirely disappeared. Shortly afterward he went to Baltimore and had a recurrent attack, from which he died. This shows what purgation will do.

Dr. M. Price: We are certainly slightly mixed in the discussion of this question. The physicians are talking about appendicitis without perforation, and the surgeons about appendicitis with perforation—conditions entirely opposite. Drs. Pepper and Meigs say they never saw but one single case of perforative appendicitis get well, and they reported that case themselves. It is true that this statement was reported fifteen years ago, but that does not make the disease any milder.

There is one other point. It is absolute folly to operate for appendicitis and expect good results, unless you can purge the patient. If you succeed, and after the operation persist in the use of purgatives, every case, so far as I know, will recover.

If at the operation there is found a barrier separating the abscess from the general peritoneal cavity, thorough irrigation of the abscess cavity is required. If this barrier cannot be demonstrated, thorough irrigation of the whole peritoneal cavity should be insisted upon. In a case operated on five weeks ago, there was well-marked thickening and induration in the right iliac fossa. I removed five or six ounces of pus, and when I came to irrigate, although I used every precaution, I found the small intestine slipping by my finger.

I washed the abdominal cavity out thoroughly, and passed in a straight glass drainage-tube, held in position by one stitch at the lower angle of the wound, and then packed with gauze down to the knuckle of intestine. The appendix was eaten off by an ulcer, and so gangrenous that I was afraid to touch it. The man was purged every day for a week, and made an uninterrupted recovery. I may mention that I have never seen a case of appendicitis with perforation and general peritoneal inflammation without a subnormal temperature.

Dr. Morton : There are many symptoms that have not been mentioned. Bladder irritation is a prominent symptom in some cases from inflammation or pressure on the ureter, or of the bladder wall. This brings out one of the dangers in operating on the appendix. Mr. Treves has predicted that some day a portion of the ureter will be taken out in mistake for the appendix or torn out with it.

It is also to be remembered that in perityphlitis the superficial veins are more engorged on the right side of the body than on the left.

Perhaps my views in regard to the time of operation have been misunderstood. What I desired to say was that no case that is not improving should be permitted to go beyond the third day without surgical interference. I do not mean to say that all cases should be left so long. In some the operation will be required in the first few hours ; in others on the first day, and still others on the second day. The great majority of cases will recover from the present attack at least upon purgation, topical applications, and regulation of diet.

Another danger of allowing septic processes to go on in the neighborhood of the appendix is the development of phlebitis in the branches of the mesenteric vein causing troubles in the liver. Pain in the liver is often a sign of appendix disease. It has been held that many cases of abscess of the liver have originated in septic processes around the appendix infecting the veins. I saw Dr. Steinbach operate on a case in which the patient before operation showed a tinge of jaundice. The appendix ran upward nearly to the liver, and surrounded by a large abscess. Intense jaundice supervened after the operation, and the man died apparently from acute inflammatory degeneration of the liver. I believe that the liver trouble was a septic complication from the appendix.

In regard to the use of calomel and podophyllin, I would say that I know very little about experimental therapeutics, but after giving the various purgatives a thorough trial I have found that minute doses of calomel and podophyllin, frequently repeated, give the best results in these cases. They move the bowels thoroughly and with rapidity. In the case reported, from fifteen to twenty movements were secured in eight or ten hours. These small doses cannot be vomited as readily as a drachm of salts can be, nor do they produce emesis even shortly after etherization, when salines would not be tolerated by the stomach. After the bowels have been thus started and the stomach quieted as a consequence, salts will be retained, if indicated, and work with greater promptitude and efficiency.

Dr. Price (closing the discussion): I rejoice that in America we have adopted some of Mr. Bryant's surgical wisdom in regard to appendicitis, as well as in regard to hernia. "If you find a man hanging, cut him down."

I will allude to three cases in which there were recurring attacks. In one, the man had had twelve to fourteen attacks. I saw him in the last in collapse on the eighth, tenth, or perhaps the twelfth day. In this case the argument offered by the family physician was the common one: that as the patient had recovered from so many attacks he would also recover from this. This is a dangerous argument and often a fatal one. I said that this man would be dead in three hours; he died in an hour and a half. In a recent case I saw the patient on Monday. Dr. Agnew saw him on the same day, and we both urged section. The physician and family decided to wait. On the following Friday, Dr. Agnew was asked to operate and refused. Many of us are now taking high ground and refusing to operate at the eleventh hour. It is not fair to surgery to operate on dying patients. Dr. Agnew has recently operated in the twenty-fifth attack, removing a huge appendix, and the boy recovered.

Deaths from appendicitis are very numerous; indeed, more so than a year ago. They were then called typhoid fever, but now our methods of diagnosis are more accurate.

Mr. Tait's recommendation of drainage has been referred to. That would be as bad surgery as to drain a huge pus tube. The cheesy, disorganized appendage remains. No one would cut down on a sequestrum in bone disease and simply put in a drainage-tube.

I have the records of two cases of appendicitis, in one of which the opening was through the lungs and the other through the esophagus. Dr. Hunter McGuire has reported a case in which the appendix was found floating in a puddle of pus. You will all remember the illustration in the *British Medical Journal* or the *Lancet* of a year ago, of a case of hepatic abscess with a large opening through the loin. You could see the liver, the kidney, and the colon. The man lived twelve days and died of dysentery. I never see a case of neglected appendicitis without thinking of this case of neglected abscess of the liver. In most cases that we see, the small intestines are enormously distended, and the pelvis is filled with adherent knuckles of bowels. In these you often have obstruction. In many cases the use of a purgative is simply folly before the adherent knuckles of intestine have been released by operation.

I scarcely favor the long-ligature method. In these cases I have inverted the stumps, and the transfixation has been made with the finest needles. If the appendix sloughs, it goes inside.

The McBurney point is wholly ununiform and worthless.

---

Paraffine in Diphtheria.—Mr. A. M. Sydney-Turner, Surgeon to the Gloucester County Infirmary, informs the *Lancet*, in reply to inquiries, that he has treated thirty cases of diphtheria (children and adults) with paraffine, and has had the satisfaction of seeing every one recover. His plan is to ask for the ordinary paraffine used in lamps, and having scraped off the diphtheritic patch, to apply the paraffine every hour to the throat (internally) with a large camel's hair brush. As a rule, the throat gets well in from twenty-four to forty-eight hours, and with improvement in the throat the paraffine is applied less frequently, but he continues to use it for two or three days after the complete disappearance of the patches. He speaks definitely as to the therapeutic effects, but is unable to state what the chemical action of paraffine on the diphtheritic membrane is; probably the hydrocarbons in the liquid exert some powerful influence on the membrane.—*Scientific American.*

---

Music and Therapeutics.—It has lately been suggested that music might prove a useful adjunct (in some cases at least) where the usual routine treatment has not been satisfactory. We venture to suggest the following airs as being suitable for the cases enumerated, viz.: Retarded labor from inertia, "Comin' Thro' the Rye;" cases of chronic deafness, "Come back to Erin;" epilepsy, "Let Me Like a Soldier Fall;" pyrexia, "The Coolin';" melancholia, "The Heart Bowed Down;" cases of doubtful diagnosis, "Oh, Dear! What Can the Matter be?"—*Medical Press.*

